# Microwave Studies of Environmental Friendly Ferroelectrics

**DOI:** 10.1155/2014/683986

**Published:** 2014-10-30

**Authors:** S. N. Mathad, Vijaya Puri

**Affiliations:** ^1^Department of Engineering Physics, KLE Institute of Technology, Hubli 580030, India; ^2^Thick and Thin Film Device Laboratory, Department of Physics, Shivaji University, Kolhapur 416004, India

## Abstract

The structural and microwave studies of lead-free barium niobates ceramics prepared by the high temperatures solid state reaction technique are reported. The structural parameters such as the lattice constants, average crystallite size (*D*), texture coefficients (TC), dislocation density, and microstrain have been determined using X-ray diffraction data. Surface morphological studies were carried out using scanning electron microscopy (SEM) technique. The strong absorption bands at ~816 cm^−1^, 641 cm^−1^, and 482 cm^−1^ are associated with the coupling mode between Nb–O stretching modes observed in FTIR studies. The electromagnetic transmittance, absorption, studies of barium niobates in the X band frequency range frequencies using waveguide reflectometer technique are reported.

## 1. Introduction

Lead-free materials are of interest as new candidates to interchange the widely used lead-based ceramics owing to their pollution free environmental friendly character throughout the preparation process. Materials that can absorb microwaves will eliminate electromagnetic radiation pollution. Wide unfolding applications of electromagnetic absorbers have affected engineers to explore relating to optimum design without their algorithms [1]. Ferroelectrics especially complex oxides with perovskite structure are inherently multifunctional materials with spontaneous polarization. The dielectric, electric, acoustic, mechanical, temperature, magnetic, and optical properties of these materials are used in a wide number of electronic applications. Components based on ferroelectric phase have a wide range of commercial applications like memory cells sensors, actuators, and so forth. Ferroelectrics in paraelectric phase have even greater potential for microwave applications. Perovskites are excellent dielectrics characterized by extraordinarily high dielectric permittivity that rely on the ferroelectric technology for microwave applications are creating their way to the industry and commercial applications, like wireless sensor networks, safety and security systems, automotive, medical, environmental food monitoring, radio tags, and so forth [2]. Good dielectrics and electric field dependent permittivity make the parametric phase ferroelectrics attractive for the development of a wide range of tunable microwave devices for applications in agile microwave systems. The materials' property from engineer's perspective, device, and system (circuit) applications of the ferroelectrics plays very important role [2]. Recent dramatic changes in microelectronics and in particular wireless communications technologies have made the importance of materials with the unusual combination of high dielectric constant, less dielectric loss, and low temperature dependence of dielectric constant of great interest. Relaxor ferroelectrics exhibit a high-dielectric constant over a wide temperature range around the ferroelectric phase transition. Ferroelectric Sr_*x*_Ba_1−*x*_Nb_2_O_6_ (0.25 ≤ *x* ≤ 0.75) with the TTB (tetragonal tungsten bronze) structure has attracted a great deal of attention and is being investigated as a potential material for pyroelectric, electro-optic, and photorefractive devices [3, 4]. Recently, it was reported that the hexagonal phase of BaNb_2_O_6_ transforms above 1200°C to the orthorhombic structure [5]. These microwave dielectrics can be synthesized by several roots syntheses like chemical methods, the coprecipitation, sol-gel, and hydrothermal and colloid emulsion techniques [5–9].

The purpose of this study was to prepare BaNb_2_O_6_ ceramics using simple low cost solid state technique from simple inorganic materials. A detailed significant investigation of the structural, microstructural, and mechanical properties of barium niobate has been reported. Electromagnetic transmittance and absorption of samples in the X band frequency range frequencies using waveguide reflectometer technique are reported.

## 2. Materials and Methods

**Figure 1:**
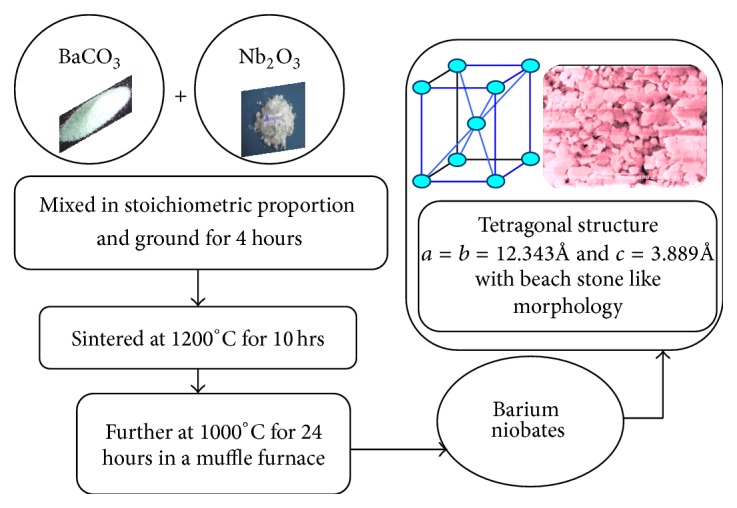
Schematic representation of the synthesis flowchart of barium niobate (BaNb_2_O_6_) ceramic.

AR grade chemicals of high purity barium carbonate (BaCO_3_ (99.95%)) and niobium penta-oxide (Nb_2_O_5_ (99.999%)) were used as starting materials. This powder was again mixed in stoichiometric proportion and ground for 4 hours in acetone medium to obtain the desired compound. This mixture was initially sintered at 1200°C for 10 hrs and further at 1000°C for 24 hours in a muffle furnace (given by (2). The flowchart of barium niobate (BaNb_2_O_6_) ceramic is shown in Figure 1:

(1) BaCO3+Nb2O5⟶BaNb2O6+CO2↑

The phase analysis was confirmed by X-ray diffraction using Cr-K_*α*_ radiations (Philips Diffract meter PW 3710). The structural parameters such as the lattice constants, average crystallite size, and texture coefficients have been determined using X-ray diffraction data. This powder was pressed into pellets in a hydraulic press at 10 ton/cm^2^ for 10 minutes. The surface morphology was studied using scanning electron microscope (SEM JEOL-JSM 6360). Transmission of microwaves due to bulk sample was measured point by point using transmission/reflection method with rectangular waveguide, consisting of the X band generator, isolator, attenuator, directional coupler, and RF detector.

### 2.1. XRD Analysis

**Figure 2:**
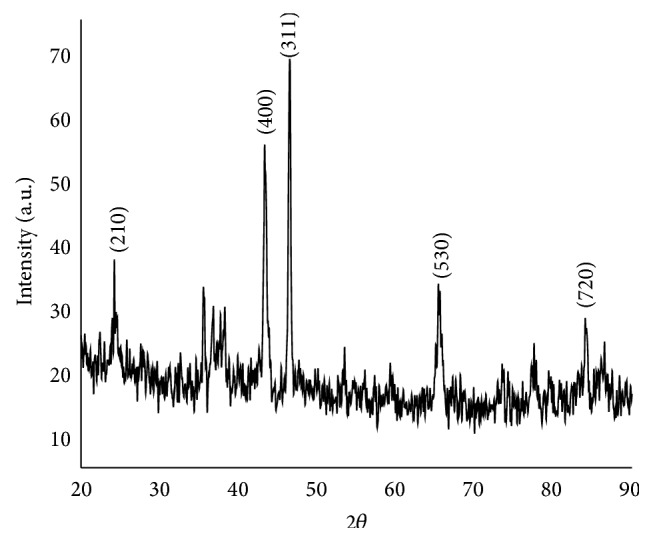
The X-ray diffraction patterns of Barium niobate.

X-ray diffraction technique is a powerful tool to analyze the crystalline nature of the materials. If the material to be investigated is crystalline, well defined peaks will be observed. X-ray diffractogram of barium niobate 2*θ* values from 20° to 90° is shown in Figure 2. The barium niobate has tetragonal structure with lattice parameters *a* = *b* = 12.34 Å and *c* = 3.89 Å (Table 1). Nanocrystalline (particle size) characteristics of the samples depend on the broadening of the XRD lines. Average particle size of the calcined powder was 37.5 nm, determined using Debye-Scherer formula [10]:

(2) $$\begin{array}{c} D=\frac{K\cdot \lambda }{\beta \cdot \cos\theta }, \end{array}$$

where *K* is Scherrer constant (*K* = 0.9), *θ* is Bragg's angle, and *β* is full-width half maxima (FWHM) in radians.

The defects distort the regular atomic array of a perfect crystal. Dislocations are 1D crystalline defects marking the boundary between slipped and unslipped regions of material. The amount of defects in the as-deposited film is assessed by dislocation density (*ρ*_*D*_). The term lattice microstrain (*ε*) is more frequently used in materials engineering. It is defined as the deformation of an object divided by its effective-length. The dislocation density (*ρ*_*D*_) and microstrain (*ε*) were calculated as [11]

(3) $$\begin{array}{c} \text{Dislocation}\;\;\text{density}\left(\rho_{D}\right)=\frac{1}{D^{2}}, \end{array}$$

(4) $$\begin{array}{c} \varepsilon =\frac{\beta \cos\theta }{4}. \end{array}$$

The Williamson-Hall equation is used to calculate the strain (*ε*) and particle size of the sample graphically given by [11, 12]

(5) $$\begin{array}{c} \beta \cos\theta =\frac{0.9\lambda }{D}+4\varepsilon \sin\theta , \end{array}$$

where *ε* is the lattice microstrain,* D* is the grain size (in Å), *λ* is the wavelength of the radiation (in Å), *θ* is the Bragg angle, and *β* is full-width half maxima (FWHM) of a XRD peak in degree.

**Figure 3:**
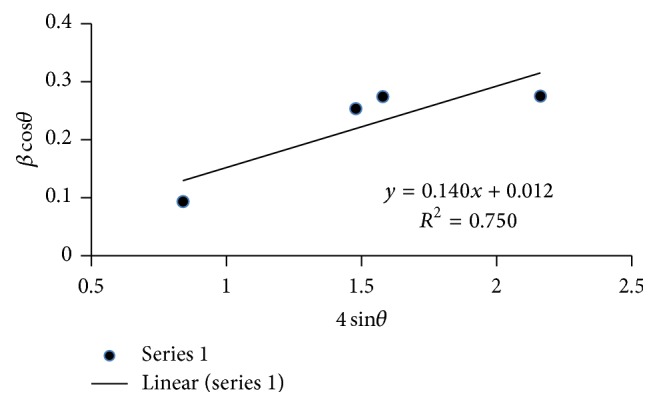
The Williamson-Hall analysis for Barium niobate.

Using a linear extrapolation fit to Williamson-Hall analysis plot (shown in Figure 3), the intercept gives the particle size (*D*) and the slope represents the strain (*ε*). Microstrain (*ε*), crystallite size (nm), and dislocation density (*D*) are tabulated in Table 2.

### 2.2. Texture Analysis

Texture is perceived in almost all engineered materials which can have a great influence on properties of materials. Diffraction patterns from samples containing a random orientation of crystallites have predictable relative peak intensities. Texture frequently represents a pole figure, in which a defined axis (crystallographic) from each of a representative number of crystallites is mapped in a stereographic projection. Texture is the distribution of crystallographic orientations of a polycrystalline sample in materials science. Quantitative data concerning the preferential crystal orientation can be obtained from the texture coefficient (TC) [13]:

(6) $$\begin{array}{c} \text{TC}\left(hkl\right)=\frac{I\left(hkl\right)/I_{0}\left(hkl\right)}{\left(1/N\right)\sum_{N}^{}\left(I\left(hkl\right)/I_{0}\left(hkl\right)\right)}. \end{array}$$

If the crystallographic orientations are fully random, then the sample has no texture. If the orientations are not random but have some preferred orientation, then the samples have a weak, moderate, or strong texture. As TC(*hkl*) increases, the preferential growth of the crystallites in the direction perpendicular to the* hkl* plane is greater (Table 3). From the texture analysis preferential grain growth which is observed at (210) plane is more dominant (TC = 2.977) and that observed at (311) plane is weak.

### 2.3. Surface Morphology

**Figure 4:**
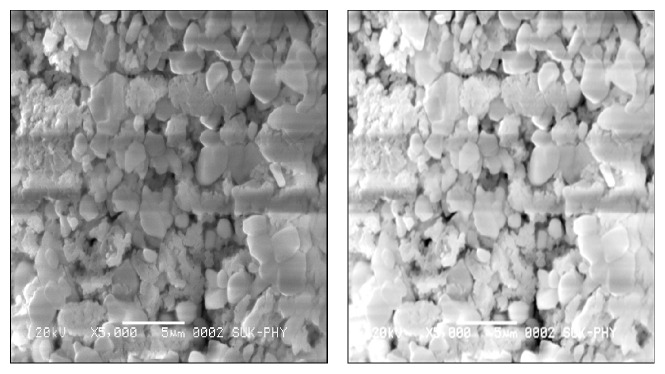
Scanning electron micrographs of barium niobate.

The morphology of barium niobate (BaNb_2_O_6_) is like typical pebble (beach stone) structure (shown in Figure 4). The image shows homogenous grains distributed over the entire volume of the samples and shows good crystallization. The average grain size varies in the range of 2–4 *μ*m. It is clear from the micrographs that the grains are densely packed in the sintered sample. However, a certain degree of porosity is still observed. The shape and distribution of grains in the microstructure exhibit the polycrystalline nature of the sample. The grain size of the sample (obtained from SEM) is larger than of the crystallite size obtained from Scherrer's equation. Thus, a single grain can be composed of several crystallites [14]. The advantages of solid-state reaction are simplicity and low cost, but the disadvantages are that the high calcining temperature results in very large grain sizes confirmed by SEM images.

### 2.4. FTIR Spectral Study

**Figure 5:**
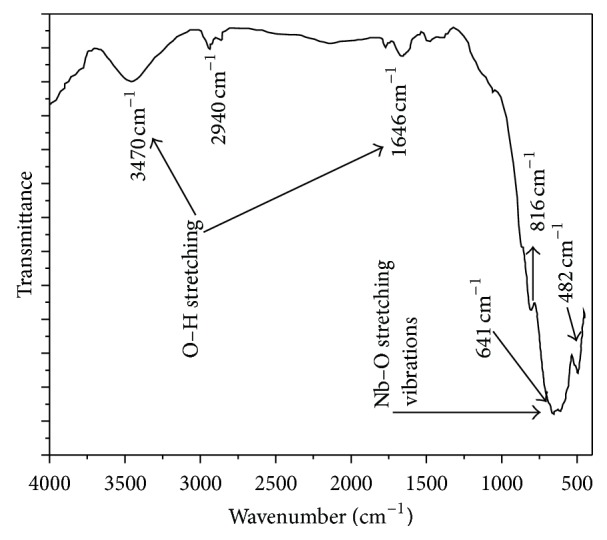
Room temperature FTIR of BaNb_2_O_6_ spectra.

The infrared spectral analysis is effectively used to understand the chemical bonding and it provides information about molecular structure of the synthesized compound. The characteristic absorption peaks in the range from 400 to 4000 cm^−1^ are shown in Figure 5. Using Perkin Elmer spectrophotometer, Fourier transform infrared (FTIR) spectra of sample (in pellet form mixed with KBr) were recorded in the range 400–4000 cm^−1^. The sharp absorption peaks at ~3470 cm^−1^ indicating the presence of hydroxide group (OH^−^) result from surface-adsorbed atmosphere (like moisture and humidity) [15]. Peaks around ~1646 cm^−1^ and 1755 cm^−1^ may be attributed to O–H bending vibrations. The strong absorption bands at ~816 cm^−1^, 641 cm^−1^, and 482 cm^−1^ are associated with the coupling mode between Nb–O stretching modes [16].

### 2.5. Microwave Studies

**Figure 6:**
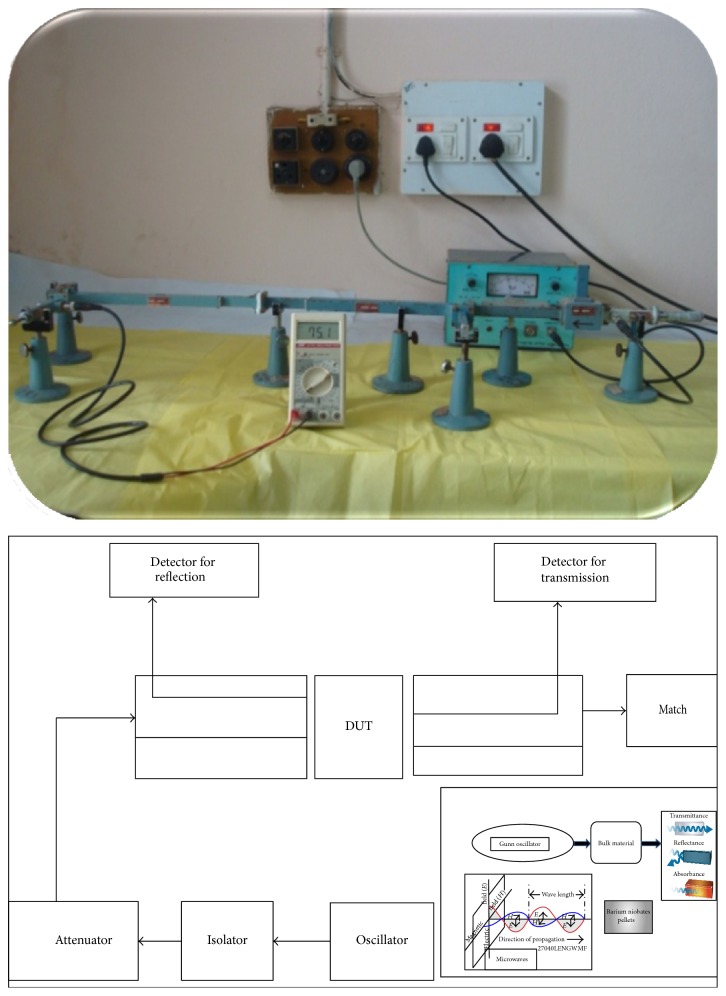
Schematic block diagram of microwave experimental setup (for transmission and reflection).

The insertion loss (transmission) and absorption loss of barium niobate sample were measured by the rectangular waveguide reflectometer setup shown in Figure 6. The microwaves were incident on the device under test (DUT) in the frequency range 8 GHz to 12 GHz (X band). The waveguide reflectometer setup consists of Gunn oscillator, isolator, attenuator, two 3 dB directional couplers connected in reverse directions, sample holder for device under test (DUT), and the diode detector. The system was calibrated by measuring the output with and without the DUT [4, 17].

**Figure 7:**
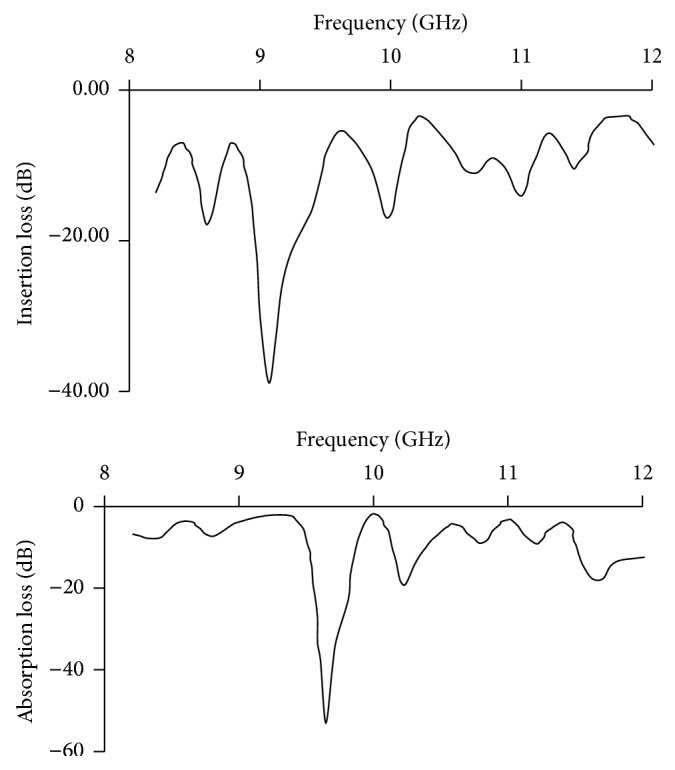
Absorption loss and insertion loss of barium niobate.

The variation of insertion loss and absorption loss of barium niobate is a function of frequency in the X band (8–12 GHz) which exhibits trivial wavy like nature shown in Figure 7. The frequency dependent variations are observed in microwave absorbance and transmittance. The average absorbance is relatively more compared to transmittance. At 9.8 GHz frequency, dip absorbance (−53 dB) may be the motion of active ferroelectric Nb^5+^↔Nb^4+^ also because the frequency of the hopping ions could not follow the applied field frequency and it lags behind.

Absorption is the heat loss under the action between electric dipole or magnetic dipole in material and the electromagnetic field. Low absorption loss in a large band of frequencies indicates potential for microwave applications [17]. These barium niobates may be used to function as sensors, actuators, detectors, filters, resonators, and so forth through special layout arrangements.

## 3. Conclusions

Lead-free ferroelectric barium niobate that has been synthesized by the solid state reaction method was investigated. It absolutely was shown by X-ray diffraction that the space temperature shows tetragonal structure, lattice parameters *a* = *b* = 12.343 Å and *c* = 3.889 Å, and average particle size was 37.5 nm with preferred (210) textured orientation. Pebble (beach stone) like morphology with grain size varied within the range of 2–4 *μ*m, confirmed by SEM. The robust absorption bands at ~816 cm^−1^, 641 cm^−1^, and 482 cm^−1^ are related to the coupling mode between Nb–O stretching modes. Microwave studies (absorbance and reflectance) depict periodical behaviour which can be used to perform like sensors, actuators, detectors, and filters.

## Figures and Tables

**Table 1:** Lattice parameter, volume, and crystallite size of BaNb_2_O_6_.

Sample	Lattice parameters			Volume(Å)^3^
	*a* (Å)	*b* (Å)	*c* (Å)	
Barium niobate	12.34	12.34	3.89	592.35

**Table 2:** Microstrain (*ε*), dislocation density (*ρ*_*D*_), and crystallite size of BaNb_2_O_6_.

Dislocation density	Strain		Crystallite size (nm)	
	From (4)	From W-H graph	From (2)	From graph
7.09 × 10^16^	0.07859	0.140	37.2	15.75

**Table 3:** Texture coefficient for significant (*hkl*) planes of BaNb_2_O_6_.

Texture coefficient for significant (*hkl*) planes	
*hkl*	TC(*hkl*)
210	2.977
400	1.386
311	0.097
530	0.187
720	0.354

## References

[1] Chamaani S., Mirtaheri S. A., Teshnehlab M., Shoorehdeli M. A., Seydi V. (2008). Modified multi-objective particle swarm optimization for electromagnetic absorber design. *Progress in Electromagnetics Research*.

[2] Gevorgian S. (2009). *Ferroelectrics in Microwave Devices: Circuits and Systems*.

[3] Neurgaonkar R. R., Cory W. K. (1986). Progress in photorefractive tungsten bronze crystals. *Journal of the Optical Society of America B: Optical Physics*.

[4] Mathad S. N., Jadhav R. N., Pawar R. P., Puri V. (2013). Electromagnetic behavior of lead free ferroelectrics at microwave frequencies. *Advanced Science, Engineering and Medicine*.

[5] Yamaguchi O., Shimizu K., Matsui K. (1985). Crystallization of hexagonal BaNb_2_O_6_. *Journal of the American Ceramic Society*.

[6] Kim D. W., Kim J. R., Yoon S. H., Hong K. S., Kim C. K. (2002). Microwave dielectric properties of low-fired Ba_5_Nb_4_O_15_. *Journal of the American Ceramic Society*.

[7] Gaikwad S. P., Samuel V., Pasricha R., Ravi V. (2004). A low temperature route to prepare BaNb_2_O_6_. *Materials Letters*.

[8] Xue J., Wan D., Lee S.-E., Wang J. (1999). Mechanochemical synthesis of lead zirconate titanate from mixed oxides. *Journal of the American Ceramic Society*.

[9] Chen G.-H., Qi B. (2006). Barium niobate formation from mechanically activated BaCO_3_-Nb_2_O_5_ mixtures. *Journal of Alloys and Compounds*.

[10] Mathad S. N., Puri V. (2012). Structural and dielectric properties of Sr_x_Ba_1-x_Nb_2_O_6_ ferroelectric ceramics. *Archives of Physics Research*.

[11] Mathad S. N., Jadhav R. N., Patil N. D., Puri V. (2013). Structural and mechanical properties of Sr^+2^-doped bismuth manganite thick films. *International Journal of Self-Propagating High-Temperature Synthesis*.

[12] Mazhdi M., Khani P. H. (2012). Structural characterization of ZnO and ZnO:Mn nanoparticles prepared by reverse micelle method. *International Journal of Nano Dimension*.

[13] Mathad S. N., Jadhav R. N., Puri V. (2012). Raman studies of Rod-like Bismuth strontium manganites. *European Journal of Applied Engineering & Scientific Research*.

[14] Allen S., Thomas E. (1999). *The Structure of Materials*.

[15] Mathad S. N., Jadhav R. N., Puri R. P. P. V. (2012). Studies on rod shaped bismuth strontium manganite ceramics. *Science of Advanced Materials*.

[16] Mathew T. V., Kuriakose S. (2013). Synthesis and characterization of sodium–lithium niobate ceramic structures and their composites with biopolymers. *Journal of Advanced Ceramics*.

[17] Jadhav R. N., Puri V. (2009). Microwave absorption, conductivity and complex pemittivity of fritless Ni_(1 − *x*)_Cu_*x*_Mn_2_O_4_(0 ≤ *x* ≥ 1) ceramic thick film: effect of copper. *Progress In Electromagnetics Research C*.

